# Clonal Hematopoiesis, Cardiovascular Diseases and Hematopoietic Stem Cells

**DOI:** 10.3390/ijms21217902

**Published:** 2020-10-24

**Authors:** Oleg Kandarakov, Alexander Belyavsky

**Affiliations:** Engelhardt Institute of Molecular Biology, Russian Academy of Sciences, 119991 Moscow, Russia; oleg.kandarakov@europe.com

**Keywords:** clonal hematopoiesis, hematopoietic stem cells, somatic mutagenesis, atherosclerosis, cardiovascular diseases, inflammatory cytokines, DNMT3A, TET2, JAK2

## Abstract

Cardiovascular diseases and cancer, the leading causes of morbidity and mortality in the elderly, share some common mechanisms, in particular inflammation, contributing to their progression and pathogenesis. However, somatic mutagenesis, a driving force in cancer development, has not been generally considered as an important factor in cardiovascular disease pathology. Recent studies demonstrated that during normal aging, somatic mutagenesis occurs in blood cells, often resulting in expansion of mutant clones that dominate hematopoiesis at advanced age. This clonal hematopoiesis is primarily associated with mutations in certain leukemia-related driver genes and, being by itself relatively benign, not only increases the risks of subsequent malignant hematopoietic transformation, but, unexpectedly, has a significant impact on progression of atherosclerosis and cardiovascular diseases. In this review, we discuss the phenomenon of clonal hematopoiesis, the most important genes involved in it, its impact on cardiovascular diseases, and relevant aspects of hematopoietic stem cell biology.

## 1. Introduction

With the advancement of medicine and decline in birth rate in industrialized countries, a substantial increase in the aging population is observed worldwide, presenting a serious challenge to healthcare systems. Incidence of cardiovascular diseases (CVD) and cancer, which are by far the most prominent causes of death or disabilities, raises exponentially with age. Accumulating evidence in recent years indicates that these pathologies may have certain common mechanisms, in particular, inflammation and somatic mutagenesis. Atherosclerosis, as a major cause of CVD, coronary artery diseases, stroke, heart failure, and venous thrombosus, is associated with inflammation and enhanced inflammatory cytokine release [1,2]. Tumorigenesis is also promoted and accelerated by inflammation [3], which is by itself a widespread phenomenon during aging [4].

The role of somatic mutagenesis and genome instability as causative factors in tumor progression has been recognized for years. However, only recently, with development of new powerful sequencing methods and bioinformatics tools, prevalence of somatic mutagenesis in aging processes has been fully acknowledged. Human aging, as now evidenced by a number of studies, is accompanied by gradual accumulation of somatic mutations in cells, in particular, stem cells responsible for tissue homeostasis in an organism. Most importantly, these studies demonstrated age-related accumulation in various tissues of cell clones containing single driver mutations, primarily in cancer-related genes. These clones are more prominent in tissues characterized by rapid proliferation and/or exposure to potentially harmful environmental factors [5,6,7]. For most of the tissues, the role of these mutant clones in aging and age-related pathological processes remains yet to be established.

## 2. Clonal Hematopoiesis

The hematopoietic system is one of the most rapidly dividing tissues in our organism, producing about 250 × 10^9^ red and white blood cells per day [8], and it is therefore no wonder that age-related somatic mutagenesis takes place in it as well. Although acquired mutations are predominantly neutral or even reduce cell fitness, some rare mutations provide selective growth advantage to cells, accelerating their proliferation, decreasing their death rate, or rendering them insensitive to growth inhibitory signals. The appearance with time of somatic blood cell clones as a result of acquisition of gene mutations conferring selective growth advantage not resulting in malignant transformation has been termed clonal hematopoiesis (CH). A more narrow term, a clonal hematopoiesis of indeterminate potential (CHIP), has been recently coined by Steensma et al. [9] to define a clonal hematopoiesis that does not result in changes of blood composition or overt hematological conditions. An alternative term for the same phenomenon, age-related clonal hematopoiesis (ARCH) [10], is used less frequently.

Historically, the first data on age-related appearance of hematopoietic clones in healthy individuals, although without knowledge of underlying somatic mutations, have been reported some 25 years ago in studies of X chromosome inactivation in women [11,12]. In these reports, involvement of hematopoietic stem cells (HSCs) in clonal expansion has been suggested on the basis of occurrence of the same patterns in various blood lineages. Later on, a notion of mutation-related clonal dominance in hematopoiesis has been developed in experiments in mice transplanted with retrovirally marked hematopoietic stem/progenitor cells (HSPCs). A number of studies demonstrated that post-transplantation hematopoiesis has been dominated by a limited number of highly expanded blood cell clones. This was attributed to the insertion of retroviral genomes in the vicinity of genes important for cell proliferation or fitness, in particular those involved in cell-cycle control, apoptosis signaling, and transcriptional regulation, which apparently provided growth advantage to these clones [13]. Surprisingly, there is essentially no overlap between the list of candidate genes that presumably induce clonal expansion in the mouse system and the genes found to be mutated in human studies of clonal hematopoiesis (described below). Although the reasons for this discrepancy are currently unknown, one might speculate that it is related to the different nature of mutagenesis mechanisms in both systems: mostly gain-of function after retroviral insertion in mice, and primarily loss-of-function by spontaneous mutagenesis in humans. Other factors, including short life-span in mice and lower resistance of their cells to transformation, might be involved as well.

Only recently, 20 years since the first reports of CH, with the advent of next-generation sequencing and related bioinformatics tools, the understanding of the nature and full scale of this phenomenon has become possible. In 2014, three studies based on exome sequencing of genes recurrently mutated in hematologic cancers reported existence of mutation-associated CH in large cohorts of individuals unselected for hematological abnormalities [10,14,15]. The majority of mutations were associated with DNMT3A (DNA methyltransferase 3 alpha), TET2 (ten-eleven translocation 2), and ASXL1 (additional sex combs-like 1) genes, whereas JAK2 (Janus kinase 2), TP53, GNAS, PPM1D, and some other genes were mutated at comparably lower frequencies. The observed mutations in CH pre-disposed affected individuals to subsequent malignant hematological transformations, increasing about 10-fold the risk of blood cancer development.

**Figure 1:**
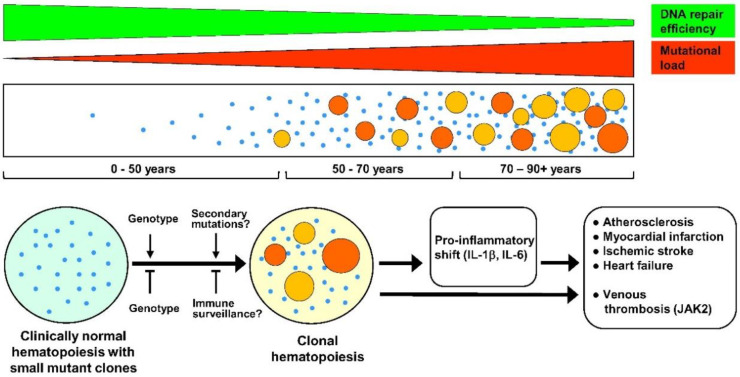
Suggested timeline of clonal hematopoiesis (CH), potential factors affecting it, and its clinical consequences. **Upper tier:** CH arises due to ongoing age-related somatic mutagenesis, which in turn is likely a result of a progressive decline of DNA repair mechanisms with aging [19,20,21,22]. **Middle tier:** age-relevant accumulation of small-size mutant clones followed by progressive appearance of the large-size ones, resulting in clinically relevant CH. **Age 0–50 years**: Cases of CH are very rare, although progressive age-dependent appearance of very small mutant clones (depicted as small blue dots) in at least some individuals is likely. **Age 50–70 years**: Very small size mutant clones are detected in 95% of the population, with usually several small clones per person [17]. In most subjects, these clones are fairly stable, and do not show many-fold expansion within 10–12 years [17]. However, in a part of the population (reaching about 20% by the age of 70 years), a strong expansion of some clones (depicted as larger-size orange and red circles) does occur, leading to a CH (defined as cases with clonal mutations greater than 0.02 variant allele fraction) with a potential clinical significance [16]. However, only 20% of these mutant clones are associated with mutations in candidate leukemia driver genes [16]. **Age 70–90+ years**: Further formation of small mutant clones is likely to occur, and large, clinically relevant mutant clones arise at increasing frequency, resulting in a more than 50% of CH prevalence in population by the age of 90 years [16]. **Bottom tier:** Although factors affecting the expansion of very small mutant clones to clinically relevant large ones are currently unknown, the genetic background must have a significant role, both in promoting and inhibiting expansion. In addition, one might presume that secondary mutations advance clonal expansion, whereas immune surveillance helps to keep mutant clones at bay. A clonal drift in the aging hematopoietic system cannot be excluded as well (not depicted). Negative effects of CH in cardiovascular diseases are gene-specific, but have a common denominator, namely pro-inflammatory shift due to enhanced production by mutant cells of inflammatory cytokines such as IL-1β (interleukin 1 beta) and IL-6 (interleukin 6). This, together with enhanced production of myeloid cells, accelerates atherosclerosis and, consequently, increases risks of myocardial infarction and ischemic stroke. These factors also contribute to cardiac fibrosis and resulting heart failure. JAK2 (Janus kinase 2) mutations, in addition, strongly enhance risks of venous thrombosis, predominantly due to increased production of thrombocytes and erythrocytes and neutrophil extracellular trap formation.

The occurrence of CH is tightly associated with age. The most clear-cut evidence for this is provided by the study of Zink et al. [16] of more than 10 thousand Icelanders using non-biased whole genome sequencing. According to their data, mutation frequency raised from a set value 0.5% in subjects younger than 35 years to nearly 50% at ages older than 85 years. Young et al. studied healthy individuals 50–70 years of age using an exquisitely accurate sequencing approach, allowing detection of very rare mutations [17]. Their results demonstrated existence of clonal hematopoiesis, frequently associated with mutations in DNMT3A and TET2 genes, in 95% of studied subjects. Earlier results reported substantially lower prevalence of CH at older ages but were based on less sensitive exome sequencing protocols [10,14,15]. Such a strikingly high prevalence of clonal hematopoiesis revealed by Young et al., combined with the finding that in most individuals, the incidence of mutations did not increase to significant values within 10–12 years, may indicate that either the genetic background exerts a strong influence on expansion of mutated clones, or that secondary mutations in other genes not analyzed in this study are required for clonal growth. Alternatively, the majority of small mutant clones may be kept in check by immune surveillance as in the case of malignant tumors [18], and concomitant with age-associated decline of the immune system, some clones may get a chance for a significant, clinically relevant expansion. A suggested timeline of CH and its relation to CVD is depicted in Figure 1.

The initial reports of CH indicated that mutations of known leukemia-associated driver genes occur in a majority of subjects with CH, but results of a more sensitive study by Zink et al. [16] demonstrate a significantly different pattern, namely that only about 20% of individuals with CH carry these leukemia-associated gene mutations. It is thus possible that the driver genes in these individuals are involved in cell growth control but have not been previously associated with hematological malignancies. Alternatively, these “unorthodox” clones may be a result of clonal drift in the aging hematopoietic system.

An important issue is a stage of hematopoietic hierarchy at which somatic mutations involved in CH arise. Initial CH reports based on X chromosome inactivation [11,12] suggested the involvement of HSCs in this phenomenon. A highly accurate sequencing study mentioned above [17] revealed that CH-associated mutations are often stable longitudinally and present in several hematopoietic lineages, which implicates multipotent long-lived HSCs as a stage affected by CH-related mutagenesis. Two other studies demonstrated that DNMT3A mutations occurred in both myeloid and lymphoid cells and thus are likely to arise in multipotent HSCs, whereas TET2 mutations were associated with myeloid compartment but not multipotent compartment [23,24]. The latter finding might indicate that TET2 mutations, in contrast to those of DNMT3A, arise at the stage of myeloid-biased or myeloid-committed HSPCs. Alternatively, TET2 mutations may occur in HSC, but impart myeloid bias on subsequent differentiation of these cells.

For the analysis of the connection of CH with CVD, the four genes, namely DNMT3A, TET2, ASXL1, and JAK2, are mostly important. These genes are discussed in more detail in the chapters below, and their potential clinical relevance is summarized in the Table 1.

## 3. DNMT3A

Among the genes whose mutations are associated with CHIP, DNMT3A demonstrates the highest mutation rate, with more than 250 mutations, affecting regulatory and catalytic domains, identified so far [10,14,15,25,26,27,28]. In general, similar mutations were identified in various hematological malignancies. DNMT3A encodes a DNA methyltransferase that is responsible for de novo introduction in DNA of the one of the most important epigenetic marks, methyl cytosine [29]. According to a study where a large cohort of patients with coronary heart disease as well as a control cohort were analyzed using whole exome sequencing, mutations in DNMT3A (as well as in TET2, ASXL1, and JAK2) were found to be associated with a significantly increased risk of coronary heart disease [30]. Association of DNMT3A mutations with CVD was also demonstrated in a number of studies [10,31,32,33]. Importantly, studies in mouse models demonstrated that inactivation of this gene in HSCs upregulates multipotency genes while suppressing genes encoding differentiation factors [34]. Dnmt3a depletion in HSCs greatly enhances their self-renewal allowing them to expand indefinitely, which manifests in their ability to support hematopoiesis after 12 cycles of serial transplantation in mice, far exceeding their normal potential of regeneration [35]. It appears that inactivation of Dnmt3a results in a pro-inflammatory shift in mice. In particular, depletion of Dnmt3a in Lin(-) bone marrow cells using CRISPR/Cas9 technology promotes cardiac Ang II-induced cardiac dysfunction [36]. Depletion of Dnmt3a in mast cells exacerbated their responses to acute and chronic stimuli [37], its deficiency also enhanced production of interferon-γ by T cells [38], and increased lung inflammation in a murine asthma model [39].

## 4. TET2

The second most frequently mutated gene in CHIP is a key enzyme that participates in an epigenetic pathway opposite to that of DNMT3A, namely active demethylation of methylcytosine in DNA. TET2 is a dioxygenase catalyzing conversion of 5-methylcytosine (5mC) into 5-hydroxymethylcytosine (5hmc) [40,41,42]. For TET2, more than 130 various mutations resulting in reduced activity of enzyme were found so far. TET2 mutations frequently occur in myelodysplastic syndromes and myeloid cancers [43,44]. Association of TET2 with CVD was demonstrated in the same studies that implicated cardiovascular connection of DNMT3A [10,30,31,32,33]. In addition, recent data indicate that patients with chronic heart failure harboring both TET2 and DNMT3A mutations are at higher mortality risk than single mutation carriers [45].

There are a number of studies demonstrating importance of murine Tet2 as a critical regulator of HSC self-renewal and differentiation in mice. Tet2 deficiency in mice results in dramatic reduction in the 5-hydroxymethylcytosine levels and concomitant increase in the 5-methylcytosine levels in bone marrow cell DNA, increased stem cell self-renewal, competitive advantage of Tet2-null HSCs over wild-type ones, progressive enlargement of the HSC compartment, and altered cell differentiation, skewing toward monocytic/granulocytic lineages with eventual myeloproliferation in vivo [46,47,48,49]. Interestingly, although the reported role of Tet2 in hematopoiesis is primarily related to its catalytic activity, it has also a non-catalytic role essential in regulation of HSPC homeostasis [50].

Important experiments revealing a connection between TET2-induced CH and atherosclerosis were performed by Fuster et al. using bone marrow transplantation of Tet2-deficient cells to atherosclerosis-prone LDLR (low density lipoprotein receptor)-deficient mice [51]. The transplanted cells competitively expanded in recipients, which led to accelerated atherosclerosis and significant increase in atherosclerotic plaque size. Moreover, TET2-null macrophages markedly increased proatherogenic IL-1β (interleukin 1 beta) secretion mediated by NLRP3 (NLR family pyrin domain containing 3) inflammasome. Essentially similar results were reported in another study with bone marrow transplantation of Tet2-null cells [30]. In this work, analysis of macrophages from Tet2 knockout mice also demonstrated enhanced expression of certain proatherogenic chemokine and cytokine genes. Depletion of Tet2 in blood cells was shown to worsen cardiac remodeling and function in two experimental murine models of heart failure, implicating a causative role of Il-1β overproduction in this process [52]. This result provides firm experimental evidence in support of epidemiological analyses connecting TET2 mutations with increased risk of cardiovascular diseases. In support of this, a recent study involving transplantation of Tet2-deficient bone marrow cells into nonirradiated mice demonstrated that transplanted animals developed age-related cardiac dysfunction due to hypertrophy and fibrosis, whereas heart macrophages had enhanced inflammatory signature [53]. In a study including a large cohort of CHIP carriers, TET2 mutations in blood cells were associated with markedly increased serum levels of IL-1β and IL-6 (interleukin 6) [54], implicating pro-inflammatory cytokines as causal factors in CVD progression connected with TET2-induced CH.

## 5. ASXL1

ASXL1 is frequently mutated in all forms of myeloid malignancies, as in the case of DNMT3A and TET2, and has a role in balancing HSC self-renewal and differentiation. ASXL1 encodes a scaffolding protein interacting with the polycomb repressing complex 2 that is involved in trimethylation of lysine 27 of histone 3 (H3K27me3) [55]. Mutations in ASXL1 are associated with increased risks of coronary heart disease and ischemic stroke [10,30].

Available data in mouse models suggest that hematopoietic-specific Asxl1 depletion reduces HSC pool, HSCs self-renewal, and hematopoietic repopulating capacity and results in multilineage cytopenias and dysplasia with features of myelodysplastic syndrome [56,57]. However, C-terminally truncated forms of Asxl1 that are similar to the most abundant ASXL1 mutations occurring in CHIP and myeloid malignancies, produce different effects, namely enlarged hematopoietic stem cell (HSC) pool and increased susceptibility to leukemic transformation [58,59,60]. No direct data as to the effects of ASXL1 mutations on atherosclerosis and cardiovascular disease are available so far. However, based on the broad similarity of phenotypes produced by C-terminally truncated forms of Asxl1 and by Dnmt3a or Tet2 depletion, similar negative effects of ASXL1 mutations on CVD pathology may be surmised.

## 6. JAK2

Association of JAK2 mutations with CH was first reported in 2005 when these mutations were detected in a vast majority of individuals with polycythemia vera (PV) [61,62], a myeloproliferative disorder characterized by overproduction of erythrocytes and thrombocytes. JAK2 mutations were also found associated at high frequency with other myeloproliferative disorders such as essential thrombocythemia and primary myelofibrosis. Therefore, mutations in this gene can be classified as CH-inducing but not CHIP-inducing ones as they produce a hematological disorder. JAK2 belongs to a family of nonreceptor tyrosine kinases transducing growth signals from type I and II cytokine receptors. In contrast to DNTM3A, TET2 and ASXL1 loss of function mutations, CH-inducing mutations in JAK2 result in a gain of function. By far the most common variant is the JAK2V671F encoding a protein with constitutive tyrosine phosphorylation activity that promotes cytokine hypersensitivity. Experiments with adoptive transfer of retrovirally transduced bone marrow cells or transgenic mouse models revealed that this mutation is sufficient for development of PV-like pathology in mice, and severity of the phenotype depends on the mouse strain and mutation load [61,63,64,65,66]. Interestingly, heterozygous expression of human JAK2V671F mutant protein in mouse knock-in model resulted in severe PV-like disease with secondary myelofibrosis, a substantially more severe pathology compared to essential thrombocythemia usual for heterozygous mutation in humans [67]. Importantly, development of PV-like pathology in the JAK2V671F transgenic model was dependent on both thrombopoietin and Stat5 (signal transducer and activator of transcription 5) signaling as inactivation of either of the genes in transgenic animals abrogated manifestations of PV [68,69], Wolach et al. found that mice with knock-in of Jak2V617F have elevated risk of thrombosis due to increased propensity for neutrophil extracellular trap formation, a component of innate immunity linked to thrombosis [70]. They also demonstrated, using a large cohort of individuals without overt myeloid disorders, that JAK2V617F-induced CH was strongly associated with an increased incidence of thrombosis.

In addition to the highly elevated risk of venous thrombotic events in carriers of JAK2V671F allele, a strikingly high, about 12-fold, increase in the risk of coronary artery disease is associated with this mutation [30], significantly higher than for mutations in three genes discussed above. This elevated risk is apparently not due to cholesterol accumulation as individuals with JAK2V671F mutation usually display lower circulating cholesterol levels [54,71]. In agreement with these findings, LDLR-knockout mice transplanted with bone marrow from JAK2V671F transgenic animals demonstrated increased atherosclerosis with early lesion formation and increased complexity in advanced state. Enhanced production of proinflammatory cytokines and chemokines, as well as cellular defects in erythrocytes and macrophages, leading to increased erythrophagocytosis, were also observed [72]. Lower blood cholesterol levels were, however, observed in mutant mice, in concordance with studies of JAK2V671F mutation in humans.

## 7. HSPC Biology and Clonal Hematopoiesis

In addition to the fact that HSPCs seem to be a major stage at which driver gene mutations result in CH, certain specific aspects of HSPC biology may be related to CH phenomenon and associated CVDs. An important issue connecting CH, HSCs, and atherosclerosis is a cholesterol metabolism. Increased cholesterol levels or defects in cholesterol efflux in HSCs has been shown to stimulate proliferation and mobilization of HSCs, and promote expansion of myeloid cells [73,74]. This is basically similar to what is found in CH, and may also exacerbate CH effects. Statins, primarily seen as drugs reducing cholesterol levels, may thus also function to inhibit excessive HSC proliferation and mitigate effects of CH, although hardly in the case of JAK2 mutations where decreased cholesterol levels were observed.

Importantly, although HSPC function appears to have significant impact on CVD pathogenesis, the opposite seems also to be true. In particular, Dutta et al. demonstrated in Apoe-/- (apolipoprotein E-deficient) mice that myocardial infarction induced proliferation of HSCs and their mobilization to spleen, which boosted monocyte production and exacerbated atherosclerotic lesions [75].

One of the potential mechanism related to CH and aging is telomere shortening, which is primarily a result of low telomerase activity and high cell proliferation rate. Accordingly, aging is accompanied by progressive shortening of telomeres in the hematopoietic system. In sequencing-based association studies, a clear association with CHIP was reported for an 8-bp deletion in the intron of the TERT gene [16,54]. Telomeres were also substantially shorter in individuals with CHIP [16]. In individuals with dyskeratosis congenita, a disorder caused by defective telomere maintenance, clonal hematopoiesis is very often, occurring in a half of patients [76]. Erosion of telomeres may eventually result in chromosome instability, paving the way to leukemogenesis. Importantly, the telomere attrition is not only caused by a high number of cell divisions, but may also be induced by oxidative stress. However, HSCs in bone marrow are located in regions of low O_2_ concentration [77,78], with the lowest oxygen concentration found in deeper peri-sinusoidal regions [79]. In addition, HCSs use aerobic glycolysis for maintaining their energy balance [80]. This, in combination with low O_2_ concentration, allows HSCs to keep reactive oxygen species (ROS) that can damage DNA at the minimal level, thus suppressing mutagenesis in HSCs. However, CH is associated with increased oxidative stress, at least as shown for myeloproliferative diseases [81,82]. The increased oxidative stress in CH may serve as a feedback loop, both stimulating mutagenesis in HSPCs and accelerating telomere attrition. However, ROS are also required for proliferation since ROS depletion below the critical level by inactivation or protein kinases AKT and AKT2 blocks HSC proliferation and differentiation [83]. Vice versa, one might expect that elevation of oxidative stress drives HSPC overproliferation independently of CH driver mutations, thus increasing the risk of CVD complications.

Another feedback loop involving HSPs is related to the pro-inflammatory shift induced by CH [84]. Recent results indicate that the pro-inflammatory environment is stimulatory for proliferation of mutated HSPCs in contrast to that of normal ones [85]. It should be noted that chronic inflammation and oxidative stress are closely related, in part, through NF-kB (nuclear factor kappa B) signaling [86]. Thus, pro-inflammatory shift and oxidative stress may reinforce each other in clonal hematopoiesis, with apparent effect on cardiovascular risks.

Finally, the role of the HSPC environment is worthy of discussion. HSPCs in bone marrow are located in specific microenvironments, called niches, likely of perivascular origin, that include endothelial cells, pericytes (mesenchymal stem cells), and some other cell components [87]. Niches of multipotent HSCs are thought to maintain them in a predominantly dormant state while allowing them to temporarily enter the cell cycle in response to physiological demand, whereas niches of more committed HSCs and progenitors are likely more permissive for proliferation and might be even promoting it. Despite the largely instructive role of niches described above, interaction of HSPCs with niches appears to be bi-directional. It is now well established that leukemic cells are able to modify their niches. Thus, MSCs in bone marrow during chronic lymphocytic leukemia proliferate slower and have features of senescent cells [88]. On the other hand, normal HSCs placed in such a leukemic niche enter the cell cycle and proliferate intensively [89]. Thus, accumulating evidence indicates the hematopoietic niches are involved in pathological processes associated with leukemic progression. Although there is currently a paucity of data regarding niche components in clonal hematopoiesis and myeloproliferative diseases, it is fairly plausible that alterations of microenvironment may have a similar, although likely less significant role in these conditions as well. In support of this hypothesis, the pro-inflammatory milieu in bone marrow of aged mice with hematological alterations reminiscent of those occurring with CH, induced changes not only in HSPCs but in their niches as well [90].

## 8. Potential Therapeutic Interventions for CH-Related Conditions

Potential treatment for TET2-mediated CH that can reduce cardiovascular risks has been suggested recently by two important studies. Agathocleous et al. [91] discovered that HSCs had unusually high levels of vitamin C (ascorbate). Vitamin C depletion in mice reduced function of Tet2, expanded the HSC pool and accelerated leukemogenesis, whereas ascorbate supplementation had the opposite effects. Ascorbate thus negatively regulates HSC function and myelopoiesis through Tet2 activation. Cimmino et al. [92] demonstrated that in mice with reversible knockdown of Tet2, ascorbate supplementation mimicked the effects of Tet2 restoration, increased the levels of 5hmc, and restored normal hematopoietic stem and progenitor cell (HSPC) self-renewal and blood homeostasis. Combination of ascorbate with decitabine resulted in higher remission rates and median overall survival I patients with acute myeloid leukemia as compared to decitabine alone, and enhanced TET2 activity in leukemic cells in vitro [93].

Since Tet2 deficiency in macrophages results in overproduction of the pro-inflammatory and pro-atherogenic cytokine IL-1β [51], accelerated plaque growth, and increased risks of heart failure in mouse models [45], this raises the possibility that the blockade of IL-1β or its receptor may be effective therapy in individuals with TET2 mutations. In support of this, targeting the interleukin-1β in the CANTOS trial with an IL-1β-neutralizing antibody canakinumab significantly lowered the risk of recurrent cardiovascular events [94]. As TET2 mutations in blood cells are associated with increased serum levels of IL-6 in addition to IL-1β [33,54], blockade of IL-6 with neutralizing antibodies might also be a promising strategy to counteract effects of TET2 mutations in HSCs.

Yet another therapeutic avenue for targeting TET2 function in hematopoiesis and CVD has been suggested by the recent study of Sun et al. [95], which demonstrated that SIRT1 (sirtuin 1) deacetylates TET2, enhancing its activity. The authors found that SIRT1 levels, and hence TET2 activity, were decreased in aberrant HSPCs in myelodysplastic syndrome (MDS), whereas overexpression of SIRT1 or its activation by agonist SRT1720 resulted in reduced myelodysplastic HSC survival. Based on these findings, synthetic SIRT1 activators or resveratrol, which is one of the potent natural compounds activating SIRT1 [96], are reasonable candidates for mitigating deleterious effects of TET2 mutations on hematopoiesis, atherosclerosis, and CVD. Consistent with this, treatment with SIRT1 agonist SRT3025 decreased levels of LDL-cholesterol and diminished atherosclerosis in Apoe(-/-) mice [97].

As for the JAK2-associated CH, Wolach et al. [70] demonstrated that treatment of thrombosis-prone Jak2V617F mice with ruxolitinib, a clinically available JAK2 inhibitor, reduced the propensity for thromb formation. Recently, Tang et al. [98] reported that selective JAK2 inhibitor TG101348 (fedratinib) treatment of Apoe-/- mice fed with high-fat high-cholesterol diet decreased phosphorylation of STAT5 and ERK1/2, reduced HSPC proliferation and excessive myelopoiesis, and resulted in substantial reduction of aortic atherosclerosis. Yang et al. [99] found that in a rabbit model of atherosclerosis based on a high-fat diet combined with aorta balloon injury, treatment with ruxolitinib substantially reduced the area of atherosclerotic plaques and inhibited production of pro-inflammatory cytokines IL-6, IL-1β, IFN-γ (interferon gamma), and TNF-α (tumour necrosis factor alpha). As in the case with TET2 mutations, blockade of IL-1β or IL-6 may also be a promising strategy for treatment of JAK2-associated MPDs. Moreover, as suggested by the studies implicating STAT5 and thrombopoietin in mutant JAK2 signaling [68,69], antagonizing these proteins may also be efficient in handling the effects of Jak2V617F mutation. Since Jak2V617F promotes thrombosis by activating β1 and β2 integrin chains in leukocytes [100], inhibition of integrin activation may be a promising strategy to reduce thrombotic CVD risks of JAK2 mutations.

Finally, bearing in mind significant advances of the recent years in cancer immunotherapy, it might be hypothesized that, for CH associated with missense mutations, it is theoretically possible that boosting the aging immune system and its immune surveillance function may lead to suppression or elimination of mutated clones. However, whether such a specific and efficient immune elimination will be clinically feasible is not currently clear.

## 9. Concluding Remarks

CH is a common condition in the elderly, and is induced by mutations in HSPCs that lead to expansion of progeny leukocytes with altered immunomodulatory properties, and to a pro-inflammatory shift inducing increase in CVD risks. Research in CH, although still quite new, already achieved important milestones in revealing the connection of CH with CVD and gene mutations involved in it.

One of the important and clinically relevant avenues in further CH research is a search for factors that contribute to expansion of clones in CH or, vice versa, may limit it. More animal experiments tailored to CH mutations combined with clinical and epidemiological studies will undoubtedly provide more precise and clinically relevant information on how CH develops and how it impacts human health in the elderly population, including potential effect of CH on non-CVD-related pathologies.

Summing up, clonal hematopoiesis, due to its relatively high incidence in an aged population and impact on leukemogenesis and CVD risks, rapidly becomes a public health issue. It appears that CH-targeted diagnostic tools must be advanced, while management of CH needs to be personalized and patient-tailored preventive care strategies elaborated.

## Figures and Tables

**Table 1:** Summary of clinical/epidemiological and experimental findings for genes with the most significant impact on clonal hematopoiesis and cardiovascular diseases.

Gene	Clinical Effects of Gene Mutations	Effects of Gene Deficiency/Mutations in Experimental Models
**DNMT3A (DNA methyltransferase 3 alpha)**	Increased risks of cardiovascular diseases, in particular: coronary heart disease, ischemic stroke, early onset myocardial infarction, coronary artery calcification. Increased risks of adverse outcomes and mortality in patients with chronic heart failure.	Upregulation of multipotency genes, strong enhancement of hematopoietic stem cell (HSC) self-renewal and expansion. Pro-inflammatory shift, cardiac hypertrophy and fibrosis, diminished cardiac function.
**TET2 (ten-eleven translocation 2)**	Increased risks of cardiovascular diseases, in particular: coronary heart disease, ischemic stroke, early onset myocardial infarction, coronary artery calcification. Increased risks of adverse outcomes and mortality in patients with chronic heart failure.	Enhancement of hematopoietic stem cell self-renewal, enlargement of the HSC compartment, myeloid shift with eventual myeloproliferation. Accelerated atherosclerosis and significant increase in atherosclerotic plaque size, proatherogenic interleukin 1β secretion in macrophages. Worsening of cardiac remodeling and function in experimental models of heart failure. Cardiac dysfunction due to hypertrophy and fibrosis, enhanced inflammatory signature in heart macrophages.
**ASXL1 (additional sex combs-like 1)**	Increased risks of coronary heart disease and ischemic stroke.	C-terminally truncated forms produce enlarged hematopoietic stem cell pool and increased susceptibility to leukemic transformation. No data as yet on effects on atherosclerosis and cardiovascular diseases.
**JAK2 (Janus kinase 2)**	Myeloproliferative diseases, polycythemia vera. Enhanced production of erythrocytes and thrombocytes. Increased risks of venous thrombosis. Increased risks of coronary heart disease.	Development of polycythemia vera-like pathology in mice, increased propensity for neutrophil extracellular trap formation. Increased atherosclerosis with early lesion formation and increased complexity in advanced state. Enhanced production of proinflammatory cytokines and chemokines, in particular interleukin 6 and interleukin 1β.

## Abbreviations

Apoe	apolipoprotein E
ARCH	age-related clonal hematopoiesis
ASXL1	additional sex combs-like 1
CH	clonal hematopoiesis
CHIP	clonal hematopoiesis of indeterminate potential
CVD	cardiovascular diseases
DNMT3A	DNA methyltransferase 3 alpha
GNAS	GNAS complex locus
HSC	hematopoietic stem cells
HSPC	hematopoietic stem/progenitor cells
IFN-γ	interferon gamma
IL-1β	interleukin 1 beta
IL-6	interleukin 6
JAK2	Janus kinase 2
LDLR	low density lipoprotein receptor
NF-kB	nuclear factor kappa B
NLRP3	NLR family pyrin domain containing 3
PPM1D	protein phosphatase, Mg2+/Mn2+ dependent 1D
PV	polycythemia vera
ROS	reactive oxygen species
SIRT1	sirtuin 1
Stat5	signal transducer and activator of transcription 5
TET2	ten-eleven translocation 2
TNF-α	tumor necrosis factor alpha
TP53	tumor protein p53

## References

[1] Swirski F.K., Nahrendorf M. (2018). Cardioimmunology: The immune system in cardiac homeostasis and disease. Nat. Rev. Immunol..

[2] Oikonomou E., Leopoulou M., Theofilis P., Antonopoulos A.S., Siasos G., Latsios G., Mystakidi V.C., Antoniades C., Tousoulis D. (2020). A link between inflammation and thrombosis in atherosclerotic cardiovascular diseases: Clinical and therapeutic implications. Atherosclerosis.

[3] Greten F.R., Grivennikov S.I. (2019). Inflammation and Cancer: Triggers, Mechanisms, and Consequences. Immunity.

[4] Libby P., Kobold S. (2019). Inflammation: A common contributor to cancer, aging, and cardiovascular diseases-expanding the concept of cardio-oncology. Cardiovasc. Res..

[5] Martincorena I., Roshan A., Gerstung M., Ellis P., Van Loo P., McLaren S., Wedge D.C., Fullam A., Alexandrov L.B., Tubio J.M. (2015). Tumor evolution. High burden and pervasive positive selection of somatic mutations in normal human skin. Science.

[6] Blokzijl F., de Ligt J., Jager M., Sasselli V., Roerink S., Sasaki N., Huch M., Boymans S., Kuijk E., Prins P. (2016). Tissue-specific mutation accumulation in human adult stem cells during life. Nature.

[7] Yizhak K., Aguet F., Kim J., Hess J.M., Kübler K., Grimsby J., Frazer R., Zhang H., Haradhvala N.J., Rosebrock D. (2019). RNA sequence analysis reveals macroscopic somatic clonal expansion across normal tissues. Science.

[8] Moore M.A.S., Morstyn G., Sheridan S. (1996). Overview of hemopoiesis and hemopoietic reconstruction. Cell Therapy: Stem Cell Transplantation, Gene Therapy and Cellular Immunotherapy.

[9] Steensma D.P., Bejar R., Jaiswal S., Lindsley R.C., Sekeres M.A., Hasserjian R.P., Ebert B.L. (2015). Clonal hematopoiesis of indeterminate potential and its distinction from myelodysplastic syndromes. Blood.

[10] Jaiswal S., Fontanillas P., Flannick J., Manning A., Grauman P.V., Mar B.G., Lindsley R.C., Mermel C.H., Burtt N., Chavez A. (2014). Age-related clonal hematopoiesis associated with adverse outcomes. N. Engl. J. Med..

[11] Fey M.F., Liechti-Gallati S., von Rohr A., Borisch B., Theilkäs L., Schneider V., Oestreicher M., Nagel S., Ziemiecki A., Tobler A. (1994). Clonality and X-inactivation patterns in hematopoietic cell populations detected by the highly informative M27 beta DNA probe. Blood.

[12] Busque L., Mio R., Mattioli J., Brais E., Blais N., Lalonde Y., Maragh M., Gilliland D.G. (1996). Nonrandom X-inactivation patterns in normal females: Lyonization ratios vary with age. Blood.

[13] Kustikova O.S., Geiger H., Li Z., Brugman M.H., Chambers S.M., Shaw C.A., Pike-Overzet K., de Ridder D., Staal F.J., von Keudell G. (2007). Retroviral vector insertion sites associated with dominant hematopoietic clones mark “stemness” pathways. Blood.

[14] Genovese G., Kähler A.K., Handsaker R.E., Lindberg J., Rose S.A., Bakhoum S.F., Chambert K., Mick E., Neale B.M., Fromer M. (2014). Clonal hematopoiesis and blood-cancer risk inferred from blood DNA sequence. N. Engl. J. Med..

[15] Xie M., Lu C., Wang J., McLellan M.D., Johnson K.J., Wendl M.C., McMichael J.F., Schmidt H.K., Yellapantula V., Miller C.A. (2014). Age-related mutations associated with clonal hematopoietic expansion and malignancies. Nat. Med..

[16] Zink F., Stacey S.N., Norddahl G.L., Frigge M.L., Magnusson O.T., Jonsdottir I., Thorgeirsson T.E., Sigurdsson A., Gudjonsson S.A., Gudmundsson J. (2017). Clonal hematopoiesis, with and without candidate driver mutations, is common in the elderly. Blood.

[17] Young A.L., Challen G.A., Birmann B.M., Druley T.E. (2016). Clonal haematopoiesis harbouring AML-associated mutations is ubiquitous in healthy adults. Nat. Commun..

[18] Swann J.B., Smyth M.J. (2007). Immune surveillance of tumors. J. Clin. Investig..

[19] Niedernhofer L.J., Gurkar A.U., Wang Y., Vijg J., Hoeijmakers J.H.J., Robbins P.D. (2018). Nuclear Genomic Instability and Aging. Annu. Rev. Biochem..

[20] Moriwaki S., Ray S., Tarone R.E., Kraemer K.H., Grossman L. (1996). The effect of donor age on the processing of UV-damaged DNA by cultured human cells: Reduced DNA repair capacity and increased DNA mutability. Mutat. Res..

[21] Goukassian D., Gad F., Yaar M., Eller M.S., Nehal U.S., Gilchrest B.A. (2000). Mechanisms and implications of the age-associated decrease in DNA repair capacity. FASEB J..

[22] Intano G.W., Cho E.J., McMahan C.A., Walter C.A. (2003). Age-related base excision repair activity in mouse brain and liver nuclear extracts. J. Gerontol. A Biol. Sci. Med. Sci..

[23] Buscarlet M., Provost S., Zada Y.F., Bourgoin V., Mollica L., Dubé M.P., Busque L. (2018). Lineage restriction analyses in CHIP indicate myeloid bias for TET2 and multipotent stem cell origin for DNMT3A. Blood.

[24] Arends C.M., Galan-Sousa J., Hoyer K., Chan W., Jäger M., Yoshida K., Seemann R., Noerenberg D., Waldhueter N., Fleischer-Notter H. (2018). Hematopoietic lineage distribution and evolutionary dynamics of clonal hematopoiesis. Leukemia.

[25] Buscarlet M., Provost S., Zada Y.F., Barhdadi A., Bourgoin V., Lépine G., Mollica L., Szuber N., Dubé M.P., Busque L. (2017). DNMT3A and TET2 dominate clonal hematopoiesis and demonstrate benign phenotypes and different genetic predispositions. Blood.

[26] Desai P., Mencia-Trinchant N., Savenkov O., Simon M.S., Cheang G., Lee S., Samuel M., Ritchie E.K., Guzman M.L., Ballman K.V. (2018). Somatic mutations precede acute myeloid leukemia years before diagnosis. Nat. Med..

[27] Acuna-Hidalgo R., Sengul H., Steehouwer M., van de Vorst M., Vermeulen S.H., Kiemeney L.A.L.M., Veltman J.A., Gilissen C., Hoischen A. (2017). Ultra-sensitive Sequencing Identifies High Prevalence of Clonal Hematopoiesis-Associated Mutations throughout Adult Life. Am. J. Hum. Genet..

[28] Abelson S., Collord G., Ng S.W.K., Weissbrod O., Mendelson Cohen N., Niemeyer E., Barda N., Zuzarte P.C., Heisler L., Sundaravadanam Y. (2018). Prediction of acute myeloid leukaemia risk in healthy individuals. Nature.

[29] Jurkowska R.Z., Jurkowski T.P., Jeltsch A. (2011). Structure and function of mammalian DNA methyltransferases. ChemBioChem..

[30] Jaiswal S., Natarajan P., Silver A.J., Gibson C.J., Bick A.G., Shvartz E., McConkey M., Gupta N., Gabriel S., Ardissino D. (2017). Clonal Hematopoiesis and Risk of Atherosclerotic Cardiovascular Disease. N. Engl. J. Med..

[31] Dorsheimer L., Assmus B., Rasper T., Ortmann C.A., Ecke A., Abou-El-Ardat K., Schmid T., Brüne B., Wagner S., Serve H. (2019). Association of Mutations Contributing to Clonal Hematopoiesis With Prognosis in Chronic Ischemic Heart Failure. JAMA Cardiol..

[32] Mas-Peiro S., Hoffmann J., Fichtlscherer S., Dorsheimer L., Rieger M.A., Dimmeler S., Vasa-Nicotera M., Zeiher A.M. (2020). Clonal haematopoiesis in patients with degenerative aortic valve stenosis undergoing transcatheter aortic valve implantation. Eur. Heart J..

[33] Bick A.G., Pirruccello J.P., Griffin G.K., Gupta N., Gabriel S., Saleheen D., Libby P., Kathiresan S., Natarajan P. (2020). Genetic Interleukin 6 Signaling Deficiency Attenuates Cardiovascular Risk in Clonal Hematopoiesis. Circulation.

[34] Challen G.A., Sun D., Jeong M., Luo M., Jelinek J., Berg J.S., Bock C., Vasanthakumar A., Gu H., Xi Y. (2011). Dnmt3a is essential for hematopoietic stem cell differentiation. Nat. Genet..

[35] Jeong M., Park H.J., Celik H., Ostrander E.L., Reyes J.M., Guzman A., Rodriguez B., Lei Y., Lee Y., Ding L. (2018). Loss of Dnmt3a Immortalizes Hematopoietic Stem Cells In Vivo. Cell Rep..

[36] Sano S., Oshima K., Wang Y., Katanasaka Y., Sano M., Walsh K. (2018). CRISPR-Mediated Gene Editing to Assess the Roles of Tet2 and Dnmt3a in Clonal Hematopoiesis and Cardiovascular Disease. Circ. Res..

[37] Leoni C., Montagner S., Rinaldi A., Bertoni F., Polletti S., Balestrieri C., Monticelli S. (2017). Dnmt3a restrains mast cell inflammatory responses. Proc. Natl. Acad. Sci. USA.

[38] Gamper C.J., Agoston A.T., Nelson W.G., Powell J.D. (2009). Identification of DNA methyltransferase 3a as a T cell receptor-induced regulator of Th1 and Th2 differentiation. J. Immunol..

[39] Yu Q., Zhou B., Zhang Y., Nguyen E.T., Du J., Glosson N.L., Kaplan M.H. (2012). DNA methyltransferase 3a limits the expression of interleukin-13 in T helper 2 cells and allergic airway inflammation. Proc. Natl. Acad. Sci. USA.

[40] Ito S., Shen L., Dai Q., Wu S.C., Collins L.B., Swenberg J.A., He C., Zhang Y. (2011). Tet proteins can convert 5-methylcytosine to 5-formylcytosine and 5-carboxylcytosine. Science.

[41] Ko M., Huang Y., Jankowska A.M., Pape U.J., Tahiliani M., Bandukwala H.S., An J., Lamperti E.D., Koh K.P., Ganetzky R. (2010). Impaired hydroxylation of 5-methylcytosine in myeloid cancers with mutant TET2. Nature.

[42] He Y.F., Li B.Z., Li Z., Liu P., Wang Y., Tang Q., Ding J., Jia Y., Chen Z., Li L. (2011). Tet-mediated formation of 5-carboxylcytosine and its excision by TDG in mammalian DNA. Science.

[43] Langemeijer S.M., Kuiper R.P., Berends M., Knops R., Aslanyan M.G., Massop M., Stevens-Linders E., van Hoogen P., van Kessel A.G., Raymakers R.A. (2009). Acquired mutations in TET2 are common in myelodysplastic syndromes. Nat. Genet..

[44] Delhommeau F., Dupont S., Della Valle V., James C., Trannoy S., Massé A., Kosmider O., Le Couedic J.P., Robert F., Alberdi A. (2009). Mutation in TET2 in myeloid cancers. N. Engl. J. Med..

[45] Cremer S., Kirschbau K., Berkowitsch A., John D., Kiefer K., Dorsheimer L., Wagner J., Rasper T., Abou-El-Ardat K., Assmus B. (2020). Multiple Somatic Mutations for Clonal Hematopoiesis Are Associated With Increased Mortality in Patients With Chronic Heart Failure. Circ. Genom. Precis. Med..

[46] Ko M., Bandukwala H.S., An J., Lamperti E.D., Thompson E.C., Hastie R., Tsangaratou A., Rajewsky K., Koralov S.B., Rao A. (2011). Ten-Eleven-Translocation 2 (TET2) negatively regulates homeostasis and differentiation of hematopoietic stem cells in mice. Proc. Natl. Acad. Sci. USA.

[47] Moran-Crusio K., Reavie L., Shih A., Abdel-Wahab O., Ndiaye-Lobry D., Lobry C., Figueroa M.E., Vasanthakumar A., Patel J., Zhao X. (2011). Tet2 loss leads to increased hematopoietic stem cell self-renewal and myeloid transformation. Cancer Cell.

[48] Quivoron C., Couronné L., Della Valle V., Lopez C.K., Plo I., Wagner-Ballon O., Do Cruzeiro M., Delhommeau F., Arnulf B., Stern M.H. (2011). TET2 inactivation results in pleiotropic hematopoietic abnormalities in mouse and is a recurrent event during human lymphomagenesis. Cancer Cell.

[49] Li Z., Cai X., Cai C.L., Wang J., Zhang W., Petersen B.E., Yang F.C., Xu M. (2011). Deletion of Tet2 in mice leads to dysregulated hematopoietic stem cells and subsequent development of myeloid malignancies. Blood.

[50] Ito K., Lee J., Chrysanthou S., Zhao Y., Josephs K., Sato H., Teruya-Feldstein J., Zheng D., Dawlaty M.M., Ito K. (2019). Non-catalytic Roles of Tet2 Are Essential to Regulate Hematopoietic Stem and Progenitor Cell Homeostasis. Cell Rep..

[51] Fuster J.J., MacLauchlan S., Zuriaga M.A., Polackal M.N., Ostriker A.C., Chakraborty R., Wu C.L., Sano S., Muralidharan S., Rius C. (2017). Clonal hematopoiesis associated with TET2 deficiency accelerates atherosclerosis development in mice. Science.

[52] Sano S., Oshima K., Wang Y., MacLauchlan S., Katanasaka Y., Sano M., Zuriaga M.A., Yoshiyama M., Goukassian D., Cooper M.A. (2018). Tet2-Mediated Clonal Hematopoiesis Accelerates Heart Failure Through a Mechanism Involving the IL-1β/NLRP3 Inflammasome. J. Am. Coll. Cardiol..

[53] Wang Y., Sano S., Yura Y., Ke Z., Sano M., Oshima K., Ogawa H., Horitani K., Min K.D., Miura-Yura E. (2020). Tet2-mediated clonal hematopoiesis in nonconditioned mice accelerates age-associated cardiac dysfunction. J.C.I. Insight.

[54] Bick A.G., Weinstock J.S., Nandakumar S.K., Fulco C.P., Leventhal M.J., Bao E.L., Nasser J., Zekavat S.M., Szeto M.D., Laurie C. (2014). Inherited Causes of Clonal Hematopoiesis of Indeterminate Potential in TOPMed Whole Genomes. bioRxiv.

[55] Itzykson R., Fenaux P. (2014). Epigenetics of myelodysplastic syndromes. Leukemia.

[56] Abdel-Wahab O., Gao J., Adli M., Dey A., Trimarchi T., Chung Y.R., Kuscu C., Hricik T., Ndiaye-Lobry D., Lafave L.M. (2013). Deletion of Asxl1 results in myelodysplasia and severe developmental defects in vivo. J. Exp. Med..

[57] Wang J., Li Z., He Y., Pan F., Chen S., Rhodes S., Nguyen L., Yuan J., Jiang L., Yang X. (2014). Loss of Asxl1 leads to myelodysplastic syndrome-like disease in mice. Blood.

[58] Balasubramani A., Larjo A., Bassein J.A., Chang X., Hastie R.B., Togher S.M., Lähdesmäki H., Rao A. (2015). Cancer-associated ASXL1 mutations may act as gain-of-function mutations of the ASXL1-BAP1 complex. Nat. Commun..

[59] Yang H., Kurtenbach S., Guo Y., Lohse I., Durante M.A., Li J., Li Z., Al-Ali H., Li L., Chen Z. (2018). Gain of function of ASXL1 truncating protein in the pathogenesis of myeloid malignancies. Blood.

[60] Nagase R., Inoue D., Pastore A., Fujino T., Hou H.A., Yamasaki N., Goyama S., Saika M., Kanai A., Sera Y. (2018). Expression of mutant Asxl1 perturbs hematopoiesis and promotes susceptibility to leukemic transformation. J. Exp. Med..

[61] James C., Ugo V., Le Couédic J.P., Staerk J., Delhommeau F., Lacout C., Garçon L., Raslova H., Berger R., Bennaceur-Griscelli A. (2005). A unique clonal JAK2 mutation leading to constitutive signalling causes polycythaemia vera. Nature.

[62] Levine R.L., Wadleigh M., Cools J., Ebert B.L., Wernig G., Huntly B.J., Boggon T.J., Wlodarska I., Clark J.J., Moore S. (2005). Activating mutation in the tyrosine kinase JAK2 in polycythemia vera, essential thrombocythemia, and myeloid metaplasia with myelofibrosis. Cancer Cell.

[63] Wernig G., Mercher T., Okabe R., Levine R.L., Lee B.H., Gilliland D.G. (2006). Expression of Jak2V617F causes a polycythemia vera-like disease with associated myelofibrosis in a murine bone marrow transplant model. Blood.

[64] Lacout C., Pisani D.F., Tulliez M., Gachelin F.M., Vainchenker W., Villeval J.L. (2006). JAK2V617F expression in murine hematopoietic cells leads to MPD mimicking human PV with secondary myelofibrosis. Blood.

[65] Tiedt R., Hao-Shen H., Sobas M.A., Looser R., Dirnhofer S., Schwaller J., Skoda R.C. (2008). Ratio of mutant JAK2-V617F to wild-type Jak2 determines the MPD phenotypes in transgenic mice. Blood.

[66] Akada H., Yan D., Zou H., Fiering S., Hutchison R.E., Mohi M.G. (2010). Conditional expression of heterozygous or homozygous Jak2V617F from its endogenous promoter induces a polycythemia vera-like disease. Blood.

[67] Marty C., Lacout C., Martin A., Hasan S., Jacquot S., Birling M.C., Vainchenker W., Villeval J.L. (2010). Myeloproliferative neoplasm induced by constitutive expression of JAK2V617F in knock-in mice. Blood.

[68] Yan D., Hutchison R.E., Mohi G. (2012). Critical requirement for Stat5 in a mouse model of polycythemia vera. Blood.

[69] Spivak J.L., Merchant A., Williams D.M., Rogers O., Zhao W., Duffield A., Resar L.S., Moliterno A.R., Zhao Z.J. (2020). Thrombopoietin is required for full phenotype expression in a JAK2V617F transgenic mouse model of polycythemia vera. PLoS ONE.

[70] Wolach O., Sellar R.S., Martinod K., Cherpokova D., McConkey M., Chappell R.J., Silver A.J., Adams D., Castellano C.A., Schneider R.K. (2018). Increased neutrophil extracellular trap formation promotes thrombosis in myeloproliferative neoplasms. Sci. Transl. Med..

[71] Liu D.J., Peloso G.M., Yu H., Butterworth A.S., Wang X., Mahajan A., Saleheen D., Emdin C., Alam D., Alves A.C. (2017). Exome-wide association study of plasma lipids in >300,000 individuals. Nat. Genet..

[72] Wang W., Liu W., Fidler T., Wang Y., Tang Y., Woods B., Welch C., Cai B., Silvestre-Roig C., Ai D. (2018). Macrophage Inflammation, Erythrophagocytosis, and Accelerated Atherosclerosis in Jak2V617F Mice. Circ. Res..

[73] Oguro H. (2019). The Roles of Cholesterol and Its Metabolites in Normal and Malignant Hematopoiesis. Front Endocrinol..

[74] Morgan P.K., Fang L., Lancaster G.I., Murphy A.J. (2020). Hematopoiesis is regulated by cholesterol efflux pathways and lipid rafts: Connections with cardiovascular diseases. J. Lipid Res..

[75] Dutta P., Courties G., Wei Y., Leuschner F., Gorbatov R., Robbins C.S., Iwamoto Y., Thompson B., Carlson A.L., Heidt T. (2012). Myocardial infarction accelerates atherosclerosis. Nature.

[76] Perdigones N., Perin J.C., Schiano I., Nicholas P., Biegel J.A., Mason P.J., Babushok D.V., Bessler M. (2016). Clonal hematopoiesis in patients with dyskeratosis congenita. Am. J. Hematol..

[77] Suda T., Takubo K., Semenza G.L. (2011). Metabolic regulation of hematopoietic stem cells in the hypoxic niche. Cell Stem Cell.

[78] Parmar K., Mauch P., Vergilio J.A., Sackstein R., Down J.D. (2007). Distribution of hematopoietic stem cells in the bone marrow according to regional hypoxia. Proc. Natl. Acad. Sci. USA.

[79] Spencer J.A., Ferraro F., Roussakis E., Klein A., Wu J., Runnels J.M., Zaher W., Mortensen L.J., Alt C., Turcotte R. (2014). Direct measurement of local oxygen concentration in the bone marrow of live animals. Nature.

[80] Takubo K., Nagamatsu G., Kobayashi C.I., Nakamura-Ishizu A., Kobayashi H., Ikeda E., Goda N., Rahimi Y., Johnson R.S., Soga T. (2013). Regulation of glycolysis by Pdk functions as a metabolic checkpoint for cell cycle quiescence in hematopoietic stem cells. Cell Stem Cell.

[81] Vener C., Novembrino C., Catena F.B., Fracchiolla N.S., Gianelli U., Savi F., Radaelli F., Fermo E., Cortelezzi A., Lonati S. (2010). Oxidative stress is increased in primary and post-polycythemia vera myelofibrosis. Exp. Hematol..

[82] Musolino C., Allegra A., Saija A., Alonci A., Russo S., Spatari G., Penna G., Gerace D., Cristani M., David A. (2012). Changes in advanced oxidation protein products, advanced glycation end products, and s-nitrosylated proteins, in patients affected by polycythemia vera and essential thrombocythemia. Clin. Biochem..

[83] Juntilla M.M., Patil V.D., Calamito M., Joshi R.P., Birnbaum M.J., Koretzky G.A. (2010). AKT1 and AKT2 maintain hematopoietic stem cell function by regulating reactive oxygen species. Blood.

[84] Cook E.K., Izukawa T., Young S., Rosen G., Jamali M., Zhang L., Johnson D., Bain E., Hilland J., Ferrone C.K. (2019). Comorbid and inflammatory characteristics of genetic subtypes of clonal hematopoiesis. Blood Adv..

[85] Abegunde S.O., Buckstein R., Wells R.A., Rauh M.J. (2018). An inflammatory environment containing TNFα favors Tet2-mutant clonal hematopoiesis. Exp. Hematol..

[86] Salminen A., Kauppinen A., Kaarniranta K. (2012). Emerging role of NF-κB signaling in the induction of senescence-associated secretory phenotype (SASP). Cell Signal..

[87] Belyavsky A.V. (2019). Niches of Hematopoietic Stem Cells in Bone Marrow. Mol Biol.

[88] Janel A., Dubois-Galopin F., Bourgne C., Berger J., Tarte K., Boiret-Dupré N., Boisgard S., Verrelle P., Déchelotte P., Tournilhac O. (2014). The chronic lymphocytic leukemia clone disrupts the bone marrow microenvironment. Stem Cells Dev..

[89] Vanegas N.P., Vernot J.P. (2017). Loss of quiescence and self-renewal capacity of hematopoietic stem cell in an in vitro leukemic niche. Exp. Hematol. Oncol..

[90] Verovskaya E., Calero-Nieto F., Reynaud D., Zhang S.Y., Herault A., Bakker S., Pietras E., Svendsen A.F., Wang X., Kinston S. (2018). Inflammatory changes in the bone marrow microenvironment drive both niche and blood system remodeling during aging. Exp. Hematol..

[91] Agathocleous M., Meacham C.E., Burgess R.J., Piskounova E., Zhao Z., Crane G.M., Cowin B.L., Bruner E., Murphy M.M., Chen W. (2017). Ascorbate regulates haematopoietic stem cell function and leukaemogenesis. Nature.

[92] Cimmino L., Dolgalev I., Wang Y., Yoshimi A., Martin G.H., Wang J., Ng V., Xia B., Witkowski M.T., Mitchell-Flack M. (2017). Restoration of TET2 Function Blocks Aberrant Self-Renewal and Leukemia Progression. Cell.

[93] Zhao H., Zhu H., Huang J., Zhu Y., Hong M., Zhu H., Zhang J., Li S., Yang L., Lian Y. (2018). The synergy of Vitamin C with decitabine activates TET2 in leukemic cells and significantly improves overall survival in elderly patients with acute myeloid leukemia. Leuk. Res..

[94] Ridker P.M., Everett B.M., Thuren T., MacFadyen J.G., Chang W.H., Ballantyne C., Fonseca F., Nicolau J., Koenig W., Anker S.D. (2017). Antiinflammatory Therapy with Canakinumab for Atherosclerotic Disease. N. Engl. J. Med..

[95] Sun J., He X., Zhu Y., Ding Z., Dong H., Feng Y., Du J., Wang H., Wu X., Zhang L. (2018). SIRT1 Activation Disrupts Maintenance of Myelodysplastic Syndrome Stem and Progenitor Cells by Restoring TET2 Function. Cell Stem Cell.

[96] Howitz K.T., Bitterman K.J., Cohen H.Y., Lamming D.W., Lavu S., Wood J.G., Zipkin R.E., Chung P., Kisielewski A., Zhang L.L. (2003). Small molecule activators of sirtuins extend Saccharomyces cerevisiae lifespan. Nature.

[97] Miranda M.X., van Tits L.J., Lohmann C., Arsiwala T., Winnik S., Tailleux A., Stein S., Gomes A.P., Suri V., Ellis J.L. (2015). The Sirt1 activator SRT3025 provides atheroprotection in Apoe-/-mice by reducing hepatic Pcsk9 secretion and enhancing Ldlr expression. Eur. Heart J..

[98] Tang Y., Liu W., Wang W., Fidler T., Woods B., Levine R.L., Tall A.R., Wang N. (2020). Inhibition of JAK2 Suppresses Myelopoiesis and Atherosclerosis in Apoe-/-Mice. Cardiovasc. Drugs Ther..

[99] Yang X., Jia J., Yu Z., Duanmu Z., He H., Chen S., Qu C. (2020). Inhibition of JAK2/STAT3/SOCS3 signaling attenuates atherosclerosis in rabbit. BMC Cardiovasc. Disord..

[100] Edelmann B., Gupta N., Schnoeder T.M., Oelschlegel A.M., Shahzad K., Goldschmidt J., Philipsen L., Weinert S., Ghosh A., Saalfeld F.C. (2018). JAK2-V617F promotes venous thrombosis through β1/β2 integrin activation. J. Clin. Investig..

